# The impact of diabetes on one-year health status outcomes following acute coronary syndromes

**DOI:** 10.1186/1471-2261-6-41

**Published:** 2006-10-24

**Authors:** Pamela N Peterson, John A Spertus, David J Magid, Fredrick A Masoudi, Kimberly Reid, Richard F Hamman, John S Rumsfeld

**Affiliations:** 1 Department of Medicine, University of Colorado at Denver and Health Sciences Center, Denver Colorado, USA; 2 Department of Medicine, Denver Health Medical Center, Denver Colorado, USA; 3 Cardiovascular Research, Mid America Heart Institute of Saint Luke's Hospital, Kansas City Missouri, USA; 4 School of Medicine, University of Missouri – Kansas City, Kansas City Missouri, USA; 5 Clinical Research Unit, Kaiser Permanente, Denver Colorado, USA; 6 Department of Preventive Medicine and Biometrics, University of Colorado at Denver and Health Sciences Center, Denver Colorado, USA; 7 Department of Medicine, VA Medical Center, Denver Colorado, USA

## Abstract

**Background:**

Diabetes is an important predictor of mortality patients with ACS. However, little is known about the association between diabetes and health status after ACS. The objective of this study was to examine the association between diabetes and patients' health status outcomes one year after an acute coronary syndrome (ACS).

**Methods:**

This was a prospective cohort study of patients hospitalized with ACS. Patients were evaluated at baseline and one year with the Seattle Angina Questionnaire (SAQ). Socio-demographic and clinical characteristics were ascertained during index ACS hospitalization. One year SAQ Angina Frequency, Physical Limitation, and Health-Related Quality of Life (HRQoL) scales were the primary outcomes of the study.

**Results:**

Of 1199 patients, 326 (37%) had diabetes. Patients with diabetes were more likely to present with unstable angina (52% vs. 40%; p < 0.001), less likely to present with STEMI (20% vs. 31%; p < 0.001), and less likely to undergo coronary angiography (68% vs. 82%; p < 0.001). In multivariable analyses, the presence of diabetes was associated with significantly more angina (OR 1.36; 95% CI 1.01–1.38), cardiac-related physical limitation (OR 1.94; 95% CI 1.57–3.24) and HRQoL deficits (OR 1.43; 95% CI 1.01–2.04) at one year.

**Conclusion:**

Diabetes is associated with more angina, worse physical limitation, and worse HRQoL one year after an ACS. Future studies should assess whether health status outcomes of patients with diabetes could be improved through more aggressive ACS treatment or post-discharge surveillance and angina management.

## Background

Acute coronary syndromes (ACS), including unstable angina, non-ST-elevation myocardial infarction (NSTEMI) and ST-elevation myocardial infarction (STEMI), affect approximately one million Americans each year [1]. As improved therapies have resulted in lower ACS mortality, patient health status has been increasingly recognized as a critical outcome, with greater emphasis being placed on the therapeutic goals of reducing symptoms, improving function, and optimizing health related quality of life (HRQoL). In addition to being an important patient-centered outcome, health status predicts subsequent mortality and morbidity in cardiac populations [2-4]. To date, few studies have examined the factors associated with health status outcomes following ACS.

Diabetes is an established and highly prevalent risk factor for cardiac morbidity and mortality, and therefore is an important comorbidity in patients with ischemic heart disease (IHD). However, little is known about the association between diabetes and health status outcomes after ACS. It is important to assess the impact of diabetes on health status outcomes in cardiovascular populations. First, there is a general perception that patients with diabetes are less likely to experience angina than patients without diabetes[5]. Yet, patients with diabetes often have a high burden of coronary atherosclerosis[6], suggesting increased potential for ischemic symptoms. To date, the severity of angina following ACS in patients with diabetes versus those without has not been described. Second, although diabetes has been associated with worse overall HRQoL in general populations [7,8], the impact of diabetes on cardiac-specific health status after ACS compared to traditional cardiac factors (e.g. ejection fraction) is not known.

The goal of this study was to prospectively evaluate the association between diabetes and cardiac-specific health status (angina frequency, physical limitation and HRQoL) in patients one year after an ACS, independent of other known predictors. The results of this study are intended to inform clinicians about the importance of diabetes as a risk factor for health status outcomes after ACS, and potentially lead to interventions to improve health status outcomes in patients with diabetes.

## Methods

### Study population

The INvestigation oF Outcomes from acute coRonary syndroMes (INFORM) study was a prospective cohort study designed to describe the health status of patients with ACS, to determine predictors of health status at one year following ACS, and to define the impact of ACS treatment on patients' health status. Patients were enrolled from two Kansas City, Missouri hospitals (Mid-America Heart Institute and Truman Medical Center) between March 1, 2001 and October 31, 2002. In total, 10,911 consecutive hospitalized patients who had a troponin blood test performed were prospectively screened for a possible ACS. Standard definitions were used to diagnose ACS patients with either myocardial infarction (MI) or unstable angina. MI patients were defined by a positive troponin blood test in the setting of symptoms or electrocardiographic changes (ST segment elevation, ST-segment depressions, or T-wave inversions) consistent with an MI[9]. Unstable angina was diagnosed if the patient had a negative troponin blood test and any one of the following: new onset angina (< 2 months) of Canadian Cardiovascular Society Classification class III or IV, prolonged (>20 minutes) rest angina, recent (< 2 months) worsening of angina, or angina that occurred within 2 weeks of an MI [10]. We excluded all potential unstable angina patients who had a diagnostic study (i.e. coronary angiography, nuclear or echocardiographic stress testing) that excluded obstructive coronary disease or who had an additional diagnostic study confirming an alternative explanation for the patients' presentation. Among the 1606 eligible patients, 1199 (75%) were enrolled. Among those who were eligible but not enrolled, 70% were discharged or transferred prior to contact, 25% refused, and 5% died prior to contact. The study received Institutional Review Board approval at both participating medical centers and all subjects signed informed consent.

Each participating patient was prospectively interviewed as early as possible during their index ACS admission to ascertain their socio-demographic and baseline health status characteristics. Detailed chart abstractions were performed to ascertain patients' medical history, laboratory results, disease severity and the processes of inpatient care. The Social Security Administration Death Master File was queried to determine patients' vital status in the year after enrollment. Surviving patients were contacted by telephone one year after ACS for a follow-up interview to reassess their clinical and health status. A minimum of 12 attempts to contact patients were made, including contacting up to 2 additional individuals designated by patients at the time of their baseline interview as people who would know their whereabouts.

### Variables/measures

Disease-specific health status was measured at baseline (during index ACS admission) and at one year post-ACS by the Seattle Angina Questionnaire (SAQ). The SAQ is a 19-item disease-specific measure for patients with coronary artery disease that has well-established validity, reproducibility, sensitivity to clinical change, and prognostic value[2,11,12]. The SAQ quantifies five clinically relevant dimensions of coronary artery disease: physical limitation, anginal stability, angina frequency, treatment satisfaction, and quality of life. The scales for these dimensions range from 0–100, with higher scores indicating fewer symptoms, better function, and better quality of life. Conversely, lower SAQ scores reflect more frequent angina, more severe physical limitation, and worse HRQoL. One-year SAQ Angina Frequency, Physical Limitation, and Quality of Life scales were the primary outcomes for this study.

To accommodate skewed distributions of SAQ scores (approximately 70% of patients had no angina symptoms at one year), and to improve scale interpretability, the SAQ angina frequency, physical limitation and HRQoL scores were dichotomized into the presence or absence of angina, presence or absence of cardiac-related physical limitation and the presence or absence of HRQoL deficits, respectively. The physical limitation section of the SAQ asks patients to indicate if they were limited due to reasons other than chest pain or angina. For analytic purposes, patients whose limitations were due to non-cardiac causes were given a score of 100 on the SAQ sub-scale (indicating no cardiac-related physical limitation). A sensitivity analysis was done excluding those indicated limitations for non-cardiac related reasons, which did not significantly alter the results. Furthermore, secondary analyses were performed using the Physical Component Summary (PCS) score from the SF-12 to evaluate the association between diabetes and overall physical health status one-year after ACS.

Overall (generic) health status was evaluated using the Short Form-12 (SF-12) health status survey. The SF-12 is reliable and valid, consisting of 12 items that reflect overall physical and mental well-being (i.e. does not contain disease-specific questions). Scoring for the PCS and Mental Component Summary (MCS) scores of the SF-12 followed the methods described by Ware et al[13]. The PCS and MCS scores range from 0–100. Higher scores on the PCS indicate fewer physical limitations, disabilities, or decrements in well-being. For the MCS, higher scores indicate frequent positive affect, less psychological distress and less limitation in usual social/role activities due to emotional problems. The one-year PCS and MCS scores were secondary outcomes in this study.

The primary independent variable of interest for this study was diabetes mellitus. Patients were considered to have diabetes if it was recorded in the medical record or if they were on diabetes medication at the time of ACS admission. A sensitivity analysis including the 44 patients newly diagnosed with diabetes during their ACS hospitalization, did not significantly alter the results. Additional variables available for risk adjustment included the demographic, cardiac factors, noncardiac factors, and treatment variables listed in Tables 1 and 2.

**Table 1:** Baseline characteristics among those with and without diabetes.

**Variables**	**Diabetes (n = 326)**	**No Diabetes (n = 873)**	**p-value**
***Demographic***			
Age, yrs (mean, SD)	62 (12)	61 (13)	0.58
Gender (% male)	51%	65%	<0.001
Race (%)			
Caucasian	72%	84%	<0.001
African American	24%	13%	
Hispanic	3%	2%	
Other	1%	1%	
***Cardiac History***			
Heart failure (%)	14%	4%	<0.001
Left ventricular ejection fraction (mean, SD)	47% (13)	47% (13)	0.82
Prior MI (%)	44%	28%	<0.001
Prior PCI (%)	38%	33%	0.11
Prior CABG (%)	24%	17%	0.01
Family history of CAD (%)	59%	55%	0.21
***Non-Cardiac History***			
Hypertension (%)	79%	61%	<0.001
Hyperlipidemia (%)	46%	33%	<0.001
History of smoking (%)	58%	70%	<0.001
Alcohol or substance abuse (%)	5%	10%	0.003
Chronic Lung Disease (%)	14%	10%	0.03
Stroke (%)	4%	1%	0.002
PVD (%)	11%	4%	<0.001
Renal Failure (%)	4%	1%	0.01
Admit Creatinine < 2.0 (%)	94%	98%	<0.001
Arthritis (%)	16%	14%	0.60

CHF = congestive heart failure; MI = myocardial infarction; PCI = percutaneous intervention; CABG = coronary artery bypass graft surgery; CAD = coronary artery disease; PVD = peripheral vascular disease

**Table 2:** ACS characteristics and treatment among those with and without diabetes.

**Variable**	**Diabetes (n = 326)**	**No Diabetes (n = 873)**	**p-value**
***ACS Characteristics***			
Type of ACS (%)			
STEMI	20%	31%	<0.001
NSTEMI	28%	29%	0.63
UA	52%	40%	<0.001
3-Vessel Disease	58%	45%	0.003
Coronary Angiography	68%	82%	<0.001
STEMI	95%	96%	
NSTEMI	84%	88%	
UA	65%	74%	
Reperfusion (acute) (%)			
PCI	18%	33%	<0.001
Thrombolytics	5%	8%	0.02
Revascularization (%)			
PCI	48%	62%	<0.001
CABG	3%	4%	
Medical Management	49%	35%	
***Discharge Treatment***			
Aspirin (%)	92%	95%	0.09
Beta-blocker (%)	79%	81%	0.41
ACE-I or ARB (%)	81%	74%	0.008
Statins (%)	75%	74%	0.94

ACS = acute coronary syndrome; STEMI = ST-elevation myocardial infarction; NSTEMI = non-ST-elevation myocardial infarction; UA = unstable angina; PCI = percutaneous intervention; CABG = coronary artery bypass graft surgery; ACE-I = angiotensin converting enzyme inhibitor; ARB = angiotensin receptor blocker

### Statistical analysis

Statistical analyses were performed using SAS version 9.1 (SAS Institute, Cary, NC). Baseline characteristics of patients with and without diabetes were compared using t-tests for continuous variables and χ^2 ^tests for categorical variables. The unadjusted associations between diabetes and primary cardiac-specific health status outcomes were evaluated by comparing the proportion of patients with any angina, any degree of physical limitation or any HRQoL deficits between those with and without diabetes using χ^2 ^tests. Secondary univariate analyses compared mean SAQ scores for each subscale and mean PCS and MCS scores one year following ACS between participants with and without diabetes using t-tests.

Multivariable logistic regression was used to evaluate the risk-adjusted association between diabetes and one-year post-ACS health status. In addition, analyses using multiple linear regression with SAQ scores as continuous outcomes were performed to ensure that categorization did not obscure any effects and yielded results similar to the primary analyses. Finally, multivariable linear regression was used to evaluate the association between diabetes and overall mental health status as measured by MCS scores from the SF-12.

Primary multivariable models were constructed using forward selection on the candidate pool of independent variables listed in Tables 1 and 2 (p < 0.10 to enter model and p < 0.05 to be retained in model), forcing in diabetes and baseline health status. For each model, the corresponding baseline health status variable was included to adjust for baseline differences in the outcome of interest. For example, models of one-year angina frequency were adjusted for baseline angina frequency. Secondary analyses were performed including all Table 1 and 2 variables as covariates in the models to maximize control of confounding.

Missing follow-up health status assessments can potentially produce a selection bias from survey non-responders that influences the results of health status studies [14]. Several sources of missing data were present in this analysis. First, 81 patients died within 12 months and thus did not have one year health assessments performed. Patients who died within 12 months were thereby excluded from analyses and the results presented should be interpreted as representative of those who survived for at least one year after ACS. Second, response data at one-year was not available for 199 patients because they could not be contacted (n = 144) or refused to complete a one-year interview (n = 55). There were no differences in the prevalence of diabetes or in baseline health status among those who responded and those who were lost to follow-up at one year. Furthermore, for those patients who refused one-year interviews or could not be contacted, propensity scores were computed using logistic regression analyses to predict their likelihood of unsuccessful follow-up. From these models, a probability of failure to complete an interview was calculated. The reciprocal of this probability was then assigned to those patients' scores in the multivariable regression analyses models in order to appropriately weight patient scores in the analysis to account for bias due to loss to follow-up. These adjustments resulted in no significant differences in effect sizes or statistical significance for the association between diabetes and angina frequency, physical limitation, or HRQoL compared to primary analyses, and thus supported no significant bias in the multivariable models.

## Results

Of the 1,199 patients in the cohort, 326 patients (27%) had diabetes. Baseline characteristics of patients with and without diabetes are listed in Table 1. Compared to those without diabetes, patients with diabetes were more likely to be female, non-Caucasian and to have a history of MI, CABG and heart failure. Hyperlipidemia, hypertension, stroke, peripheral vascular disease, renal dysfunction and chronic lung disease were also more common among those with diabetes. There was no significant difference among those with and without diabetes in left ventricular function.

ACS characteristics and treatments are presented in Table 2. Patients with diabetes were more likely to present with unstable angina (52% vs. 40%; p < 0.001) and less likely to present with STEMI (20% vs. 31%; p < 0.001). Patients with diabetes were less likely to have coronary angiography performed during index ACS hospitalization (68% vs. 82%; p < 0.001). The proportion of participants with and without diabetes receiving angiography was significantly different among those with unstable angina (65% vs. 74%), but similar among those with NSTEMI (84% vs. 88%) and STEMI (95% vs. 96%). Among patients who underwent coronary angiography, those with diabetes were more likely to have 3-vessel coronary artery disease (58% vs. 45%; p = 0.003). In the year following the index ACS hospitalization, there was no significant difference between those with and without diabetes in the number of percutaneous interventions performed (16.5% vs. 20%; p = 0.82). There was a trend toward more CABG procedures (12% vs. 6%; p = 0.06) in patients with diabetes.

At the time of the index hospitalization, patients with diabetes had lower mean SAQ scores for angina frequency (72 vs 77; p = 0.001) and physical limitation (78 vs 87; p < 0.001), but no significant difference in HRQoL scores (49 vs 50; p = 0.25). One year following ACS, patients with diabetes had significantly lower SAQ angina frequency (85 vs. 91; p = 0.0006), physical limitation (82 vs. 92; p < 0.0001) and HRQoL (76 vs. 84; p < 0.0001) scores compared to patients without diabetes.

**Figure 1:**
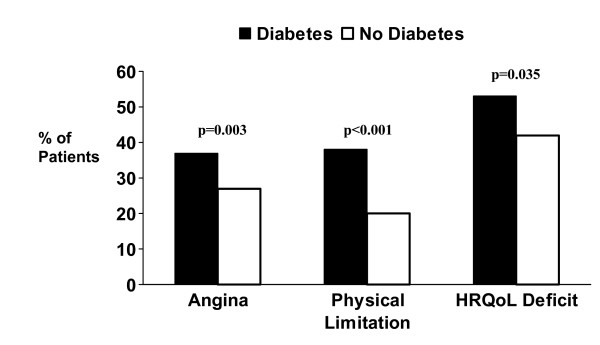
Unadjusted proportions of patients with and without diabetes with angina, cardiac-specific physical limitations and HRQoL deficits as measured by the SAQ one year following ACS.

In unadjusted analysis, patients with diabetes, when compared to those without diabetes, were significantly more likely to have angina of any degree (37% vs. 27%; p = 0.003), cardiac-related physical limitation (35% vs. 27%; p < 0.001), and HRQoL deficits (53% versus 42%; p = 0.035, Figure 1) one year following ACS. Furthermore, patients with diabetes had worse overall physical health status (mean PCS scores 37 vs. 44; p < 0.0001), and mental health status (mean MCS scores 52 vs. 55; p = 0.0013) compared to patients without diabetes.

In multivariable analyses, diabetes remained significantly associated with an increased presence of angina, cardiac-related physical limitation and HRQoL deficits one year after ACS. After adjustment for demographic, cardiac, non-cardiac, and treatment variables, patients with diabetes had significantly higher odds of having angina (OR 1.36; 95% CI 1.01–1.38), cardiac-related physical limitation (OR 1.94; 95% CI 1.57–3.24) and HRQoL deficits (OR 1.43; 95% CI 1.01–2.04) at one year compared to patients without diabetes.

In multivariable models, additional predictors of having angina were age (OR 1.08; 95% CI 1.00–1.15 per decade decrement), history of MI (OR 1.60; 95% CI 1.21–2.13), hypercholesterolemia (OR 0.75; 95% CI 0.57–0.98), history of alcohol or substance abuse (OR 2.0; 95% CI 1.20–3.38), chronic lung disease (OR 2.74; 95% CI 1.76–4.26), and history of peripheral vascular disease (OR 1.75; 95% CI 1.02–3.02). Additional predictors of physical limitation were female gender (OR 1.65; 95% CI 1.19–2.28), Caucasian race (OR 0.61; 95% CI 0.40–0.93), history of MI (OR 1.61; 95% CI 1.15–2.24), history of alcohol or substance abuse (OR 2.28; 95% CI 1.27–4.09), discharge prescription of an angiotensin converting enzyme inhibitor or an angiotensin receptor blocker (OR 1.52; 95% CI 1.07–2.16) and chronic lung disease (OR 2.15; 95% CI 1.15–4.00). The additional predictors of HRQoL deficits were age (OR 1.28; 95% CI 1.14–1.45 per decade decrement), ejection fraction (OR 1.12 (1.01–1.27 per 10% decrease), acute thrombolytic therapy (OR 1.92; 95% CI 1.01–3.66), and chronic lung disease (OR 2.36; 95% CI 1.33–4.20).

In multivariable linear regression analyses, diabetes remained significantly associated with worse overall physical function as measured by the SF-12. PCS scores were, on average, three points lower for those with diabetes compared to those without diabetes (β = 2.85 +/- 1.12, p = 0.01). Diabetes was not independently associated with differences in MCS scores (β = 1.26 +/- 0.93, p = 0.18).

## Discussion and conclusion

The goal of this study was to examine the association between diabetes and patients' health status outcomes one year after an ACS. We found that patients with ACS and coexisting diabetes were more likely to present with unstable angina, and were less likely to undergo coronary angiography during ACS hospitalization. At one year following ACS, patients with diabetes were significantly more likely to have angina, cardiac-related physical limitation and HRQoL deficits compared to patients without diabetes. These associations remained significant after adjustment for a wide array of demographic, disease severity, comorbid condition, and treatment variables. Diabetes was also negatively associated with overall physical health status, but was not significantly associated with overall mental health status.

It may be surprising that patients with diabetes were more likely to have angina one-year post ACS. Previous studies have shown that patients with diabetes have a higher rate of silent myocardial ischemia [15-17], although this has not been a consistent finding [18,19]. However, it is not appropriate to conclude that diabetics with coronary artery disease necessarily experience less angina. To our knowledge, no previous study has directly evaluated the angina burden of patients with diabetes after ACS. The results of this study support that patients with diabetes are more likely than non-diabetics to have angina one year after ACS.

The results of this study support the potential for more aggressive identification and treatment of angina in patients with diabetes following ACS in order to improve outcomes. Angina decreases patients' quality of life, is associated with worse patient satisfaction, and increases the risk for subsequent ACS and death[2,3,20-22]. There are a range of therapies for angina, including pharmacologic management, coronary revascularization, and other interventions such as enhanced external counterpulsation [23]. However, angina is often inadequately recognized and treated in routine clinical practice, including under-use and under-dosing of beta-blockers, long acting nitrates, and calcium channel blockers [24-27]. Future studies should assess whether the post-ACS outcomes of patients with diabetes can be improved by employing strategies to enhance post-discharge surveillance and treatment of angina such as disease management or cardiac rehabilitation. While evidence supports these interventions in general cardiac populations, [28,29] studies have not specifically targeted patients with diabetes.

We also found that patients with diabetes and unstable angina were less likely to receive coronary angiography, raising the possibility that greater use of an early invasive strategy in diabetic patients could improve outcomes. The discrepancy in angiography rates did not appear to be wholly or directly attributable to clinician concerns over impaired renal function given that 94% of diabetic patients had admission creatinine levels <2.0 μg/ml. Current American Heart Association/American College of Cardiology guidelines for ACS state that in the absence of high-risk indicators such as new ST-segment depression, symptoms of heart failure or high-risk findings on non-invasive testing, an early conservative *or *invasive approach can be taken with similar morbidity and mortality outcomes [30]. Given the results of this study, in which diabetics are more prone to persistent angina and diminished quality of life after an ACS, future clinical trials should assess whether an early invasive approach in patients with diabetes presenting with ACS, even in the absence of other high-risk indicators, can improve health status outcomes.

The results of this study expand the previous literature on diabetes and health status in cardiac populations. Prior studies have suggested that diabetes is associated with greater overall physical limitation and worse quality of life following CABG surgery or PCI [31,32]. Previous studies on the association between diabetes and post-ACS health status outcomes have been conflicting [33,34]. Importantly, studies to date have not included evaluation of cardiac-specific health status, and the absence of baseline measures of health status in most of these studies precluded the ability to adjust for baseline health status differences. This study therefore adds to our knowledge by prospectively evaluating the influence of diabetes on the health status of patients with ACS, using both cardiac-specific and generic measures of health status in a large unselected population of patients across the spectrum of ACS, including adjustment for baseline health status.

Potential limitations to this study should be considered. First, missing follow-up health status assessments can bias quality of life studies [14]. However, there was no difference in the rate of missing surveys between patients with and without diabetes. Furthermore, statistical methods to assess for the impact of missing values did not suggest significant bias in the multivariable models. Second, as an observational study, residual unmeasured confounding may be present. However, a wide range of measured variables were considered in multivariable regression models. One potential confounder, severity of CAD, is difficult to quantify in patients with diabetes, who are known to be at risk for more diffuse CAD. In this study, severity of CAD was adjusted for as possible using the combination of 1, 2, or 3-vessel coronary artery disease and left ventricular ejection fraction. Finally, because the focus of the parent study was ACS and not diabetes per se, details on diabetic microvascular complications were not collected and could not be specifically considered in multivariable models. However, renal function, peripheral vascular disease and baseline health status were included, which are likely to reflect pre-existing diabetic complications influencing health status.

In conclusion, we found that diabetes was associated with more angina, greater cardiac-specific physical limitation, and worse HRQoL one year after an ACS. Future studies should evaluate interventions to reduce angina burden and improve the health status of patients with diabetes following ACS. Potential strategies include an early invasive approach for unstable angina in patients with diabetes and more aggressive surveillance and therapy for angina in the post-ACS period. It is hoped that such interventions will optimize the patient-centered outcomes of symptom burden, physical function, and quality of life for the growing population of patients with diabetes and CAD.

## Competing interests

1) Dr. Frederick Masoudi serves on the speaker's bureau of AstraZeneca, the speaker's bureau of Takeda NA and the advisory board of Takeda NA. Dr. John Spertus serves as a consultant for CV Therapeutics; and 3) Dr. John Rumsfeld served as a consultant for CV Therapeutics and Pfizer.

## Authors' contributions

PP participated in the design of the study, performed statistical analyses and drafted the manuscript. JS participated in the study design and coordination and helped draft the manuscript. DM, FM, RH and JR participated in the design of the study helped draft the manuscript. KR performed statistical analyses and helped draft the manuscript. All authors read and approved the final manuscript.

## Pre-publication history

The pre-publication history for this paper can be accessed here:


